# Recurrent Drug-Induced Hypersensitivity Syndrome Following Bortezomib for Multiple Myeloma

**DOI:** 10.7759/cureus.20830

**Published:** 2021-12-30

**Authors:** Austin B Ambur, Rajiv Nathoo

**Affiliations:** 1 Dermatology, Advanced Dermatology and Cosmetic Surgery, Oviedo, USA

**Keywords:** proteasome inhibitors, multiple myeloma, drug-induced hypersensitivity syndrome, bortezomib, dress syndrome

## Abstract

Drug-induced hypersensitivity syndrome is a rare, severe, and delayed hypersensitivity reaction that may occur with exposure to a number of medications. Typical implicated medications include aromatic anticonvulsants, sulfonamides, minocycline, dapsone, and allopurinol. Bortezomib is a proteasome inhibitor and has rarely been associated with cutaneous hypersensitivity reactions. We report a case of recurrent drug-induced hypersensitivity syndrome secondary to bortezomib in a patient with multiple myeloma. The aim of this article is to highlight a unique mediation that may cause drug-induced hypersensitivity syndrome and to emphasize the challenge of managing these patients long-term to prevent relapse of the syndrome.

## Introduction

Bortezomib is a proteasome inhibitor that is utilized in the treatment of multiple myeloma. Common adverse effects of bortezomib include dizziness, lightheadedness, nausea, vomiting, loss of appetite, and fatigue. Recent literature has demonstrated an association of various severe cutaneous adverse reaction (SCAR) syndromes with the use of this medication, including drug-induced hypersensitivity syndrome (DIHS), toxic epidermal necrolysis, and Sweet’s syndrome [1-3]. DIHS is characterized by an extensive cutaneous eruption, visceral organ involvement, lymphadenopathy, eosinophilia, and atypical lymphocytes. We report a case of DIHS secondary to bortezomib in a patient with multiple myeloma. The diagnosis may be life-threatening which makes early recognition and treatment essential. Therapeutic management of this condition presents its own challenge as relapse is common. This article was previously presented as a meeting abstract at the 2019 AAD (American Academy of Dermatology) Annual Meeting on March 11, 2019.

## Case presentation

**Figure 1:**
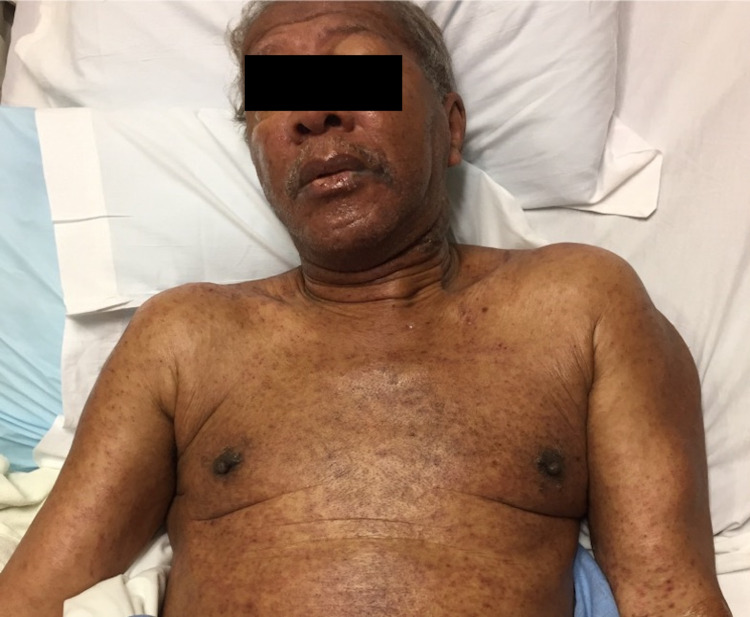
At initial presentation, diffuse erythematous to hyperpigmented patches with some overlying excoriation on the trunk, bilateral upper extremities, and face. Erosions on the upper and lower mucosal lip.

**Figure 2:**
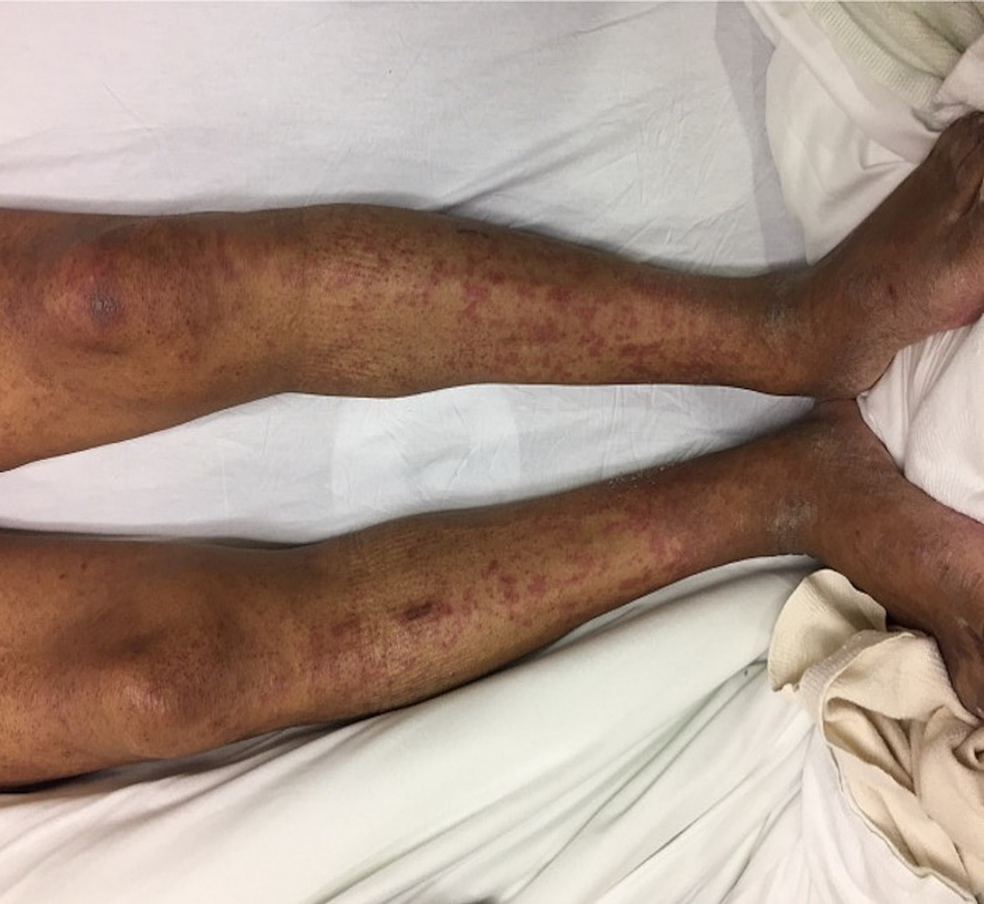
At initial presentation, diffuse erythematous to hyperpigmented patches with some overlying excoriation on the bilateral lower extremities.

A 70-year-old African American male with a history of multiple myeloma that was treated with lenalidomide and bortezomib was admitted to the hospital two weeks after his first bortezomib infusion. The patient presented with a fever, hypereosinophilia, elevated liver function enzymes and decreased renal function. Blood culture, hepatitis panel, and urinalysis were negative for infection. Physical examination was remarkable for diffuse erythematous and hyperpigmented patches with some overlying excoriation on the trunk, bilateral upper extremities, and face. Erosions were noted on the upper and lower mucosal lip (Figures 1, 2).

**Figure 3:**
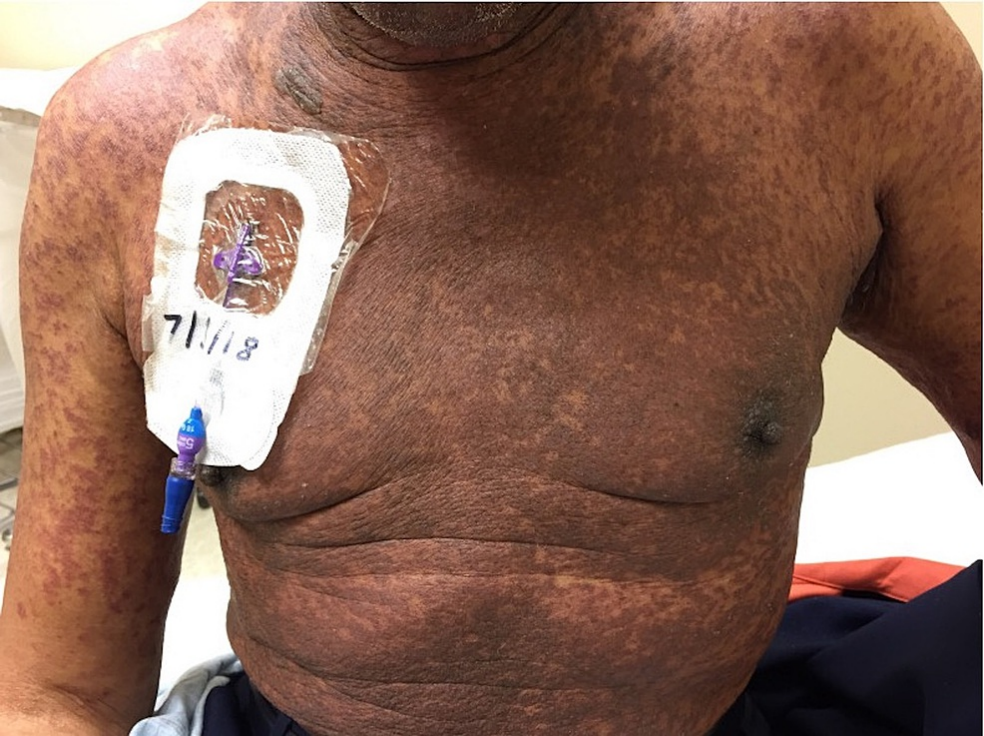
At 2 weeks following initial presentation, diffuse and symmetric erythematous to violaceous scaly papules and plaques, coalescing on the chest with islands of sparing.

**Figure 4:**
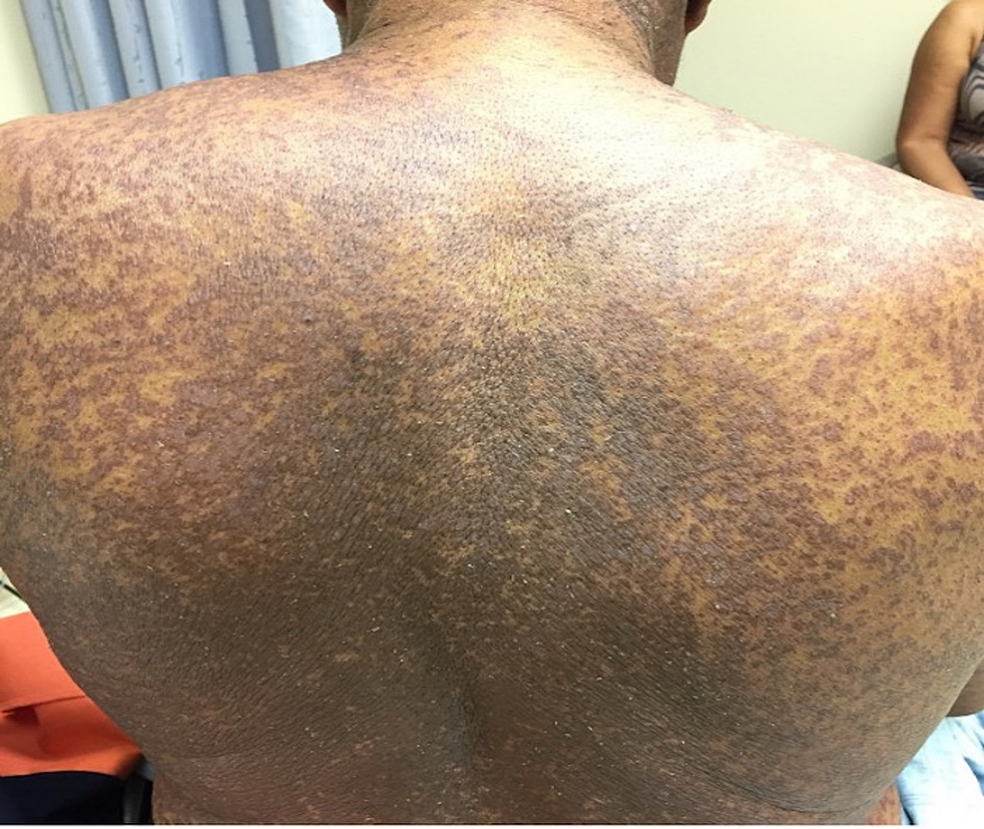
At 2 weeks following initial presentation, diffuse and symmetric erythematous to violaceous scaly papules and plaques, coalescing on the back with islands of sparing.

The calculated RegiSCAR (severe cutaneous adverse drug reaction) score was 4, suggesting probable DRESS (drug rash with eosinophilia and systemic symptoms) syndrome. Given the onset of symptoms, bortezomib was determined to be the most likely culprit. Medication reconciliation was unremarkable for any alternative causes of the reaction. Chemotherapy was held, and the patient was started on methylprednisolone (1 g twice a day) for three days. He was transitioned to an oral steroid taper and discharged on prednisone (60 mg daily). His rash, mucositis, itching, and malaise initially improved. However when he was transitioned to prednisone 40 mg daily, one week after discharge, the rash progressively worsened and was associated with a decline of liver and renal function. The patient was admitted and noted to have a worsening diffuse, erythematous-violaceous, scaly, 10/10 pruritic rash (BSA 80%) with intact vesicles scattered on the volar arms (Figures 3, 4).

Laboratory work demonstrated a creatinine of 2.3, an ALT (alanine transaminase) of 203 (from 323), and an AST (aspartate transferase) of 72 (from 150). The RegiSCAR score was now 5, given that more than 50% of body surface area was involved, suggesting possible DIHS/DRESS. Three punch biopsies demonstrated lichenoid dermatitis, which could have been interpreted as drug-induced dermatitis. However, the histopathological changes associated with DIHS are not well-established, and the patient’s systemic involvement was more suggestive of DIHS [4]. The patient was discharged on prednisone (80 mg/day) and outpatient triamcinolone 0.01% ointment wraps. He experienced an improvement in the rash and pruritus with a gradual taper over six weeks.

## Discussion

DIHS is characterized as a delayed hypersensitivity reaction that may occur after exposure to various medications. The latency period is prolonged and typically occurs two to eight weeks after drug exposure. Common clinical features include fever, rash, lymphadenopathy, facial edema, hematologic abnormalities, and internal organ involvement. The liver is the most common internal organ involved, however, other gastrointestinal manifestations such as colitis, esophagitis, and pancreatitis may occur [5,6]. Pulmonary involvement may manifest as an interstitial infiltrate and pleural effusions and is frequently misdiagnosed as pneumonia [7]. Myocarditis is an under-recognized manifestation of DIHS which may progress to acute necrotizing eosinophilic myocarditis [8]. The visceral involvement associated with DIHS may be associated with fatal complications and requires close long-term monitoring. Diagnostic lab abnormalities include atypical lymphocytes, eosinophilia, mild elevation in liver enzymes, and renal abnormalities. The most commonly implicated medications include aromatic anticonvulsants, sulfonamides, minocycline, dapsone, and allopurinol [9]. Although the cause is largely unknown, there is a potential for human herpesvirus 6 (HHV-6), human herpesvirus 6 (HHV-7), cytomegalovirus (CMV), and Epstein-Barr virus (EBV) reactivation [10]. Recent reports have shown it to develop after the use of bortezomib [2]. This chemotherapeutic agent is a proteasome inhibitor that acts through inhibition of the 26S proteasome, thereby preventing the activation of NF-kB. The end result of this inhibition is apoptosis of rapidly dividing atypical cells [11]. Treatment of DIHS includes stopping the causative medication and beginning systemic steroids. Management of these patients can be challenging because relapse of the condition is common if steroids are tapered too rapidly. It is essential for the clinician to counsel patients on this risk and to stress the importance of patient compliance with the long steroid taper to avoid recurrent DIHS. Intravenous immunoglobulins, cyclosporine, tofacitinib, and cyclophosphamide have been utilized as second-line therapies in severe cases of DIHS.

## Conclusions

We report a case of drug-induced hypersensitivity syndrome secondary to bortezomib that re-occurred upon steroid taper in a patient with multiple myeloma to highlight a rare cause of the syndrome. We also use this case to emphasize the temporal relationship of DIHS to aid in the search for causality. Clinicians should be aware of the potential for refractory DIHS if steroid therapy is tapered too quickly.
